# A step towards long-wavelength protein crystallography: subjecting protein crystals to a vacuum

**DOI:** 10.1107/S1600576715006147

**Published:** 2015-05-09

**Authors:** Santosh Panjikar, Lars Thomsen, Kane Michael O’Donnell, Alan Riboldi-Tunnicliffe

**Affiliations:** a Australian Synchrotron, 800 Blackburn Road, Clayton, VIC 3168, Australia; b Department of Physics, Astronomy and Medical Radiation Physics, Curtin University, Bentley, WA 6102, Australia

**Keywords:** lysozyme, proteinase K, vacuum, long-wavelength crystallography, sulfur single-wavelength anomalous diffraction

## Abstract

Using the UHV experimental endstation on the soft X-ray beamline at the Australian Synchrotron, lysozyme and proteinase K crystals have been exposed to a vacuum of 10^−5^ mbar, prior to flash-cooling in a bath of liquid nitrogen. Subsequent data collection on the MX2 beamline at the Australian Synchrotron demonstrated that, for lysozyme and proteinase K, it is possible to subject these mounted crystals to a vacuum pressure of 10^−5^ mbar without destroying the crystal lattice. Despite the lower data quality of the vacuum-pumped crystals compared with control crystals, it is demonstrated that the protein crystals can survive in a vacuum under suitable conditions.

## Abstract 2

Protein crystals have been subjected to a vacuum pressure of 10^−5^ mbar prior to flash-cooling, and data acquired at either 7 or 13 keV.

## 1. Introduction   

It is relatively easy to produce proteins for crystallization experiments, and the use of robotics has allowed a wider range of crystallization space to be sampled quickly and efficiently. Furthermore, owing to improvements at synchrotron sources and in detector technology, diffraction experiments can now be performed on ever smaller crystals. However, there is still a bottleneck in solving structures which have no (or not enough) similarity to existing structures in the Protein Data Bank (http:/www.rscb.org; Berman *et al.*, 2000 ▶) to allow the structure to be determined by molecular replacement. These cases will require structure solution to be carried out by experimental phasing methods (*i.e.* heavy-atom soaks, Se–Met incorporation *etc.*) which are often time-consuming.

Phasing experiments using sulfur as a scatterer were first undertaken in the early 1980s (Hendrickson & Teeter, 1981 ▶). Since then, improvements in synchrotron radiation sources and detectors have allowed the determination of many structures using this method.

In order to maximize the anomalous differences of these intrinsic elements, one must use X-rays with a very long wavelength (the sulfur edge at 2.485 keV gives ∼4 electrons, as does phosphor at 2.173 keV). As these wavelengths are ‘unreachable’ on current macromolecular crystallography beamlines, data are more often collected in the region of 4–7 keV (3–1.77 Å). However, at these wavelengths air absorption becomes a real problem. To date, either the air absorption problem has been ignored or attempts to overcome the absorption effect have been undertaken by replacing the air path with a helium path. This entails affixing a ‘sock’ reaching from the sample to the detector. In order to maintain the helium atmosphere, the sample needs to be cooled using a helium cryostat (a piece of equipment that is expensive, as is the use of helium).

This system obviously has limitations, *i.e.* the detector cannot easily be moved with a ‘sock’ bolted onto the front, and it is expensive to run. More commonly, the air absorption problem has been ignored, meaning only well diffracting crystals have been amenable to phasing from sulfur single-wavelength anomalous diffraction (S-SAD).

However, recent work has shown the possibility of using multiple crystal averaging as an approach for structure determination using S-SAD, and this process has been demonstrated on conventional-sized crystals (Liu *et al.*, 2012 ▶, 2013 ▶, 2014 ▶; Omari *et al.*, 2014 ▶) and using an X-ray free-electron laser (X-FEL) (Barends *et al.*, 2013 ▶) at energies between 6 and 7 keV.

Diamond Light Source (Didcot, UK) is currently commissioning a beamline designed specifically for long-wavelength crystallography and has removed the air absorption/scatter problem by removing the air path completely by holding the crystal in a vacuum. In this paper, we show a proof of principle that protein crystals (lysozyme and proteinase K) can survive in a vacuum of 10^−5^ mbar (1 bar = 100 000 Pa) for 5 min or more without suffering damage through loss of solvent. To the best of our knowledge, these are the first experiments where diffraction patterns have been collected on *hydrated* crystals subjected to a vacuum. Earlier work was undertaken by Perutz & Rogers (1946 ▶) in the 1940s in which *dehydrated* crystals were subjected to X-ray diffraction experiments in a specially built vacuum tank. The elimination of air scattering allowed Bragg reflections from haemoglobin crystals to be observed, which were otherwise lost in the background.

## 2. Materials and methods   

Lyophilized powders of lysozyme and proteinase K (purity ≥90%) were purchased from Sigma–Aldrich. Crystals of lysozyme suitable for this investigation were obtained under ‘standard’ conditions (50 mg ml^−1^ solution in 0.1 *M* sodium acetate trihydrate pH 4.6, mixed in a ratio of 1:1 with a reservoir solution containing 1.0 *M* NaCl and 0.1 *M* sodium acetate trihydrate pH 4.6), as were proteinase K crystals (20 mg ml^−1^ in 50 m*M* HEPES pH 7.0, mixed in a ratio of 1:1 with a reservoir of 1.2 *M* ammonium sulfate and Tris–HCl pH 8.0). Four crystals of lysozyme were mounted on four different 20 µm diameter nylon loops and coated with a layer of Paratone oil. In the text below they are referred to as follows:

(1) 30 min Control. The crystal was looped out of mother liquor from a drop and a thick coating of Paratone-N was applied. It was left at atmospheric pressure for 30 min, whereafter it was immediately flash-cooled.

(2) Control 1. The crystal was looped out of mother liquor from a drop and cryo-protected by Paratone-N, whereafter it was immediately flash-cooled.

(3) Vacuum Thick. The crystal was looped out of mother liquor from a drop and a thick coating of Paratone-N was applied. It was then subjected to a vacuum of 1.5 × 10^−5^ mbar for 5 min, whereafter it was immediately flash-cooled.

(4) Vacuum Thin. The crystal was looped out of mother liquor from a drop and a thin coating of Paratone-N was applied. It was then subjected to a vacuum of 1.5 × 10^−5^ mbar for 5 min, whereafter it was immediately flash-cooled.

For proteinase K, only one crystal was mounted on a 20 µm diameter nylon loop and coated with a layer of Paratone-N oil. The proteinase K diffraction data statistics and structure were compared with a standard proteinase K structure from the PDB (comparison data not shown).

The crystals were taken to the soft X-ray experimental endstation vacuum chamber and inserted into the loadlock chamber, which has a 360 l s^−1^ Leybold turbo pump attached as well as an Edwards XDS10 scroll pump, allowing us to evacuate the atmosphere to a pressure of 1.5 × 10^−5^ mbar within a few minutes. Subsequently, the chamber was vented to atmospheric pressure with nitrogen, whereafter the samples were immediately removed from the vacuum chamber and flash-cooled in liquid nitrogen. The lysozyme Control 1 crystal was mounted, flash-cooled and used immediately in a diffraction experiment. The second reference sample (30 min Control) was kept at atmospheric pressure on the bench for 30 min (the amount of time it took to loop, coat with oil, transfer to the soft X-ray chamber, mount on sample holders, and pump down the Vacuum Thin and Vacuum Thick crystals). All crystals were flash-cooled in a bath of liquid nitrogen prior to transfer to the MX2 beamline for data collection.

The data used for structure determination of all the crystals were collected on an ADSC Q315r CCD diffractometer attached to the 3ID1 beamline at the Australian Synchrotron, using monochromatic radiation of 7 keV for the lysozyme crystals and 13 keV for the proteinase K crystal. All crystals were held in a stream of nitrogen gas at 100 K. Diffraction data were processed using *XDS* (Kabsch, 2010 ▶) and reduced using *Scala* (Evans, 1993 ▶). S-SAD phasing was successful for the Vacuum Thick data, but difficult for the given quality of the Vacuum Thin data. Therefore, the structures of all the crystals were solved by the molecular replacement method (McCoy *et al.*, 2007 ▶; Vagin & Teplyakov, 2000 ▶) within *Auto-Rickshaw* (Panjikar *et al.*, 2005 ▶), with further refinement using *REFMAC5* (Vagin *et al.*, 2004 ▶) from the *CCP4* suite of programs (Winn *et al.*, 2011 ▶).

## 3. Results   

Results are shown in the tables of data-collection statistics (Table 1 ▶) and refinement statistics (Table 2 ▶) for the crystals investigated in this manuscript.

## 4. Discussion   

We have demonstrated that it is possible to subject mounted lysozyme and proteinase K crystals to vacuum pressures of 10^−5^ mbar without destroying the crystal lattice. When undertaking subsequent diffraction experiments post vacuum subjection, we see some deterioration of the diffraction and data quality of the crystals. It should be stressed that the crystals have been under a vacuum and brought back to atmospheric pressure, whereafter they were flash-cooled. The crystals survived in a vacuum because of the layer of oil over them. The use of organic cryo-protectants such as glycerol or ethylene glycol destroyed the crystals after vacuum exposure. There are two reasons why our vacuum-exposed and oil-covered crystals lost diffraction quality: firstly the change from a vacuum to atmospheric pressure, and secondly the de­hydration of the crystals in oil while under a vacuum or atmospheric pressure.

The two proteins we have used in these experiments, lysozyme and proteinase K, have solvent contents of ∼37 and ∼46%, respectively. A greater change in the unit cell of the protein crystal with the higher solvent content (proteinase K) was expected when exposed to a vacuum. However, this is not the case, as we see a shift for lysozyme in the *c* axis of the unit cell from the standard ∼37 Å observed in both of the control samples and the Vacuum Thick sample to a smaller ∼34 Å for the Vacuum Thin sample, while no significant change is observed for the proteinase K crystal exposed to a vacuum compared with the control sample (PDB code 2g4v; Mueller-Dieckmann *et al.*, 2007 ▶).

**Figure 1:**
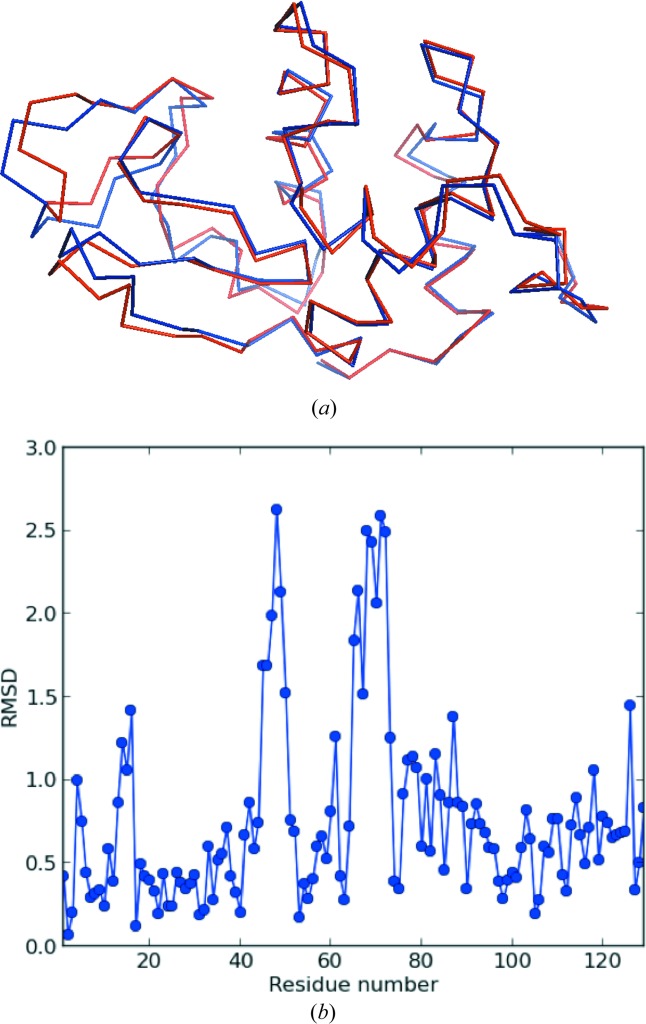
(*a*) Blue denotes the Cα chain of the 30 min Control sample and red that of the Vacuum Thin sample. The main areas of movement are found in the loops containing residues 43–50 and residues 64–73. (*b*) Plot of RMSD of the Cα chains between the 30 min Control and Vacuum Thin samples.

Even though the crystals were coated in Paratone-N, the Vacuum Thin lysozyme sample did lose solvent whilst under a vacuum (the change in the unit cell was not observed in either of the control crystals nor the Vacuum Thick crystal), and this loss of solvent caused a change in the overall shape of the monomer, with the two lobes coming closer together, as seen in Fig. 1 ▶(*a*).

The Vacuum Thin and control lysozyme crystals were compared and the average root mean-square deviation (RMSD) over 129 residues was 1.4 Å. Significant RMSDs were observed between residues 43 and 50, and between residues 64 and 73 (Fig. 1 ▶ *b*). These regions belong to the two lobes and point towards the solvent channel in the crystal packing.

In the experiment presented here, we had to allow the samples to return to atmospheric pressure prior to cooling and during data acquisition. This has an impact on the diffraction and data quality of the crystals. However, if the diffraction from an oil-coated crystal could be recorded under a vacuum at room temperature, relatively good quality data might be collected. Keeping the crystals cold under a vacuum could provide further advantages, with a reduction in radiation damage, an increase in the lifetime of the protein crystals and an improvement in data quality.

## 5. Summary   

We have successfully shown that hydrated lysozyme and proteinase K protein crystals can survive exposure to a vacuum of 10^−5^ mbar. These results show promise for long-wavelength experiments in crystallography.

## Figures and Tables

**Table 1:** Data-collection statistics Values after a solidus (/) are for the high-resolution shell.

	Lysozyme				
Data	30min Control	Control 1	Vacuum Thick	Vacuum Thin	Proteinase K
Wavelength ()	1.7712	1.7711	1.7712	1.7711	0.9537
Total frames	360	360	360	360	360
Oscillation	1	1	1	1	1
Space group	*P*4_3_2_1_2	*P*4_3_2_1_2	*P*4_3_2_1_2	*P*4_3_2_1_2	*P*4_3_2_1_2
Unit cell (, )	*a* = 78.97	*a* = 78.18	*a* = 76.77	*a* = 76.15	*a* = 67.52
	*b* = 78.97	*b* = 78.18	*b* = 76.77	*b* = 76.15	*b* = 67.52
	*c* = 36.91	*c* = 37.00	*c* = 36.83	*c* = 34.33	*c* = 102.59
	= 90.00	= 90.00	= 90.00	= 90.00	= 90.00
	= 90.00	= 90.00	= 90.00	= 90.00	= 90.00
	= 90.00	= 90.00	= 90.00	= 90.00	= 90.00
Resolution ()	1.8	1.8	1.8	2.22	1.91
Mosaicity ()	0.062	0.189	0.281	0.283	0.195
Total reflections	286640/37496	279557/37193	259501/32934	135977/20210	474867/61496
Unique reflections†	20574/3265	20292/3249	19526/3128	9542/1507	35182/5645
Redundancy	13.93/11.48	13.78/11.45	13.29/10.53	14.25/13.41	13.50/10.89
Completeness (%)	99.1/97.5	99.8/98.8	99.7/98.4	99.6/97.4	99.9/99.5
Mean *I*/(*I*)	24.28/15.42	29.57/10.22	27.65/13.25	20.88/4.07	47.16/22.35
*R*_merge_ (%)‡	9.8/11.9	6.7/17.7	7.7/15.3	8.3/64.4	5.3/10.6
*R*_meas_ (%)‡	10.2/12.4	6.9/18.5	8.0/16.0	8.6/66.9	5.5/11.1
Wilson *B*	17.82	21.59	22.79	47.99	14.25

† Friedel pairs kept separate.

‡ *R*_merge_ = *_hkl_* *_i_*|*I_i_*(*hkl*) *I*(*hkl*)|/*_hkl_* *_i_I_i_*(*hkl*) and *R*_meas_ (*R*_r.i.m._) = *_hkl_*[*N*/(*N* 1)]^1/2^_*i*_|*I_i_*(*hkl*) *I*(*hkl*)|/*_hkl_* *_i_I_i_*(*hkl*), where *I_i_*(*hkl*) is the *i*th intensity measurement of reflection *hkl*, *I*(*hkl*) is its average and *N* is the redundancy of a given reflection.

**Table 2:** Refinement statistics

	Lysozyme				
Data	30min Control	Control 1	Vacuum Thick	Vacuum Thin	Proteinase K
Resolution range ()	201.80	201.80	201.80	202.8	201.91
Reflections used for refinement (all)	10216	10084	9703	2733	17993
Reflections used for *R*_free_	988	983	949	123	1030
*R*_cryst_ †	18.9	19.5	17.4	24.9	14.1
*R*_free_	23.8	23.1	20.7	30.9	18.7
R.m.s. deviation for bonds ()	0.023	0.022	0.024	0.012	0.025
R.m.s. deviation for angles ()	1.811	1.832	1.961	1.585	1.871
Residues in most favoured region (%)	87.6	86.7	85.8	71.65	89.8
Residues in additional allowed region (%)	12.4	13.3	14.2	14.96	9.8
Residues in generously allowed regions (%)	0	0	0	13.39	0.4
Disallowed region (%)	0	0	0	0	0

† Statistics are based on *PROCHECK* (Laskowski *et al.*, 1993 ▶).
